# Electrochemical Reduction of Amine-Captured Carbon Dioxide Catalyzed by Transition-Metal Substituted Polyoxometalates

**DOI:** 10.3390/molecules31183276

**Published:** 2026-09-16

**Authors:** Dima Azaiza-Dabbah, Ronny Neumann

**Affiliations:** Department of Molecular Chemistry and Materials Science, Weizmann Institute of Science, Rehovot 76000, Israel; azaiza.dima@weizmann.ac.il

**Keywords:** electrocatalysis, polyoxometalate, carbon dioxide, carbamate, polyethylenimine

## Abstract

The low temperature electrocatalysis of CO_2_ to CO typically requires purified CO_2_, adding complexity, cost, and energy penalties due to the need for separate CO_2_ capture and purification processes. Electrochemical reactive capture (e-RCC) is emerging as a simplification of the process. The method combines CO_2_ capture with amines to form ammonium carbamates which are then reduced to produce CO and H_2_. Heterometallic polyoxometalate catalysts were evaluated for e-RCC by cyclic voltammetry showing that [SiCu_2_^II^Ga^III^(H_2_O)_3_W_9_O_37_]^9−^ had the lowest overpotential and highest turnover frequency. A combination of cyclic voltammetry and controlled potential electrolysis and transport measurements of carbamates through Nafion membranes revealed that commonly used small-molecule carbamates easily traversed membranes and were oxidized at the anode to CO and CO_2_. To avoid anodic amine oxidation, polyammonium carbamates were prepared, and the method provides an efficient way to convert CO_2_ captured into valuable carbon monoxide. Controlled potential electrolysis confirmed that [SiCu_2_^II^Ga^III^(H_2_O)_3_W_9_O_37_]^9−^ reduced polyammonium carbamate at a negative potential of −1 V vs. Ag/AgCl, forming CO and H_2,_ while other polyoxometalate catalysts yield CO and H_2_ at more negative potentials of −1.3 V. This research combines the use of polyoxometalates for electrocatalysts for e-RCC and provides a potential pathway to avoid detrimental amine/carbamate anodic oxidation.

## 1. Introduction

The rising concentration of CO_2_ in Earth’s atmosphere is a critical factor in the ongoing challenges of climate change and sustainability [1]. Among the strategies for converting excess CO_2_ into valuable chemicals is the low-temperature electrochemical reduction of CO_2_ (e-CO_2_RR) [2,3], where advances toward commercialization have been made using nanoparticle catalysts [4]. Concurrently to the use of metal nanoparticle catalysts for e-CO_2_RR, homogeneous transition-metal catalysis has also been broadly investigated following earlier seminal research by the groups of Lehn, Saveant, and Kubiak among many others [5,6,7]. Molecular catalysts may have advantages in active site design and control and spectroscopic elucidation of reaction intermediate, especially toward the two-electron reduced products CO and formate [8].

Typically, e-CO_2_RR requires the use of a relatively high purity CO_2_ feedstock, and therefore e-CO_2_RR needs to be coupled to carbon capture and utilization (CCU) technologies that still are challenging particularly in terms of high costs, energy efficiency, and the technological maturity required to scale these solutions [9]. The need for high-purity CO_2_ for e-CO_2_RR has spurred more recent investigations into the possibility of utilizing low-purity CO_2_ for e-CO_2_RR either by integrating CCU and e-CO_2_RR methods yielding so-called electrochemically reactive capture (e-RCC) systems [10,11] or similar development of direct e-CO_2_RR [12]. Although the study of integrated e-RCC systems has not yet matured [13,14], they have the perceived and calculated advantage of reducing the overall cost of CO_2_ reduction through the elimination of costs related to the formation of high-purity CO_2_ [15,16,17,18,19]. In addition, scenarios predict an advantage through an increase in cathodic CO_2_ concentrations thereby potentially increasing faradaic efficiencies and product yields.

Two concepts have been investigated toward e-RCC reactions. The first relies on the formation of bicarbonate using a hydroxide base. Since no catalyst has been reported to directly electrochemically reduce bicarbonate to two-electron products such as CO or formate, the concept relies on in situ release of CO_2_ by protons formed by the anodic four-electron oxidation of water [20,21,22,23]. It is reported that the pH at the cathode surface significantly influences the product selectivity, and pH control is crucial for obtaining reactivity [24,25]. The second pathway is based on the broadly used method of CO_2_ capture with primary and secondary amines, notably diethanolamine (DEA), monoethanolamine (MEA), and methyldiethanolamine (MDEA), among others, to yield mostly corresponding carbamates. In typical CO_2_ purification units, the carbamates are thermally decomposed back to CO_2_ and amine [26,27,28]. In e-RCC reactions the carbamates formed in situ have been shown to be reduced on various cathode materials to yield mostly the two-electron reduced CO and formate, along with significant amounts of H_2_ [10,11,29,30,31,32,33,34,35,36,37,38,39,40,41,42,43,44,45]. Despite the advances made, product selectivity, reduced Faradaic efficiencies (FE) compared the reactions without amines, higher H_2_ yields through the competing hydrogen evolution reaction (HER), and corrosion of metallic catalysts under reducing potentials all remain significant challenges toward viable e-RCC technology. Importantly, it should be noted that in all the e-RCC reactions described in the literature, the formation of CO and/or CO_2_ by anodic oxidation of small molecule amines and/or the corresponding carbamates has not been considered. This is certainly the case for reactions carried out in undivided cell configurations, but also for the divided cell configuration (H-cell or electrolyzers) where the permeability of small-molecule amines and/or the corresponding carbamates through proton exchange membranes (PEMs) or anion exchange membranes (AEMs) has not been considered.

In our laboratory we have recently investigated the use of transition-metal substituted polyoxometalates for ambient temperature e-CO_2_RR. For example, [SiW_9_Cu_3_(H_2_O)_3_O_37_]^10–^ anions were shown to be highly effective catalysts, >95% FE, for the selective electrocatalytic reduction of CO_2_ gas to CO in acetonitrile, albeit at rather negative potentials, −2.5 V vs. Fc/Fc^+^ [46]. More recently, it was shown that the introduction of a Lewis acid into the polyoxometalate framework, notably Ga(III), yielding [SiW_9_Cu^II^Fe^III^Ga^III^(H_2_O)_3_O_37_]^8–^, can lead to electrocatalytic reduction of CO_2_ to CO at low overpotentials of only 250–300 mV [47]. Thus, it was of interest to see if similar polyoxometalate compounds would be active catalysts toward CO formation in e-RCC reactions of carbamates.

**Figure 1:**
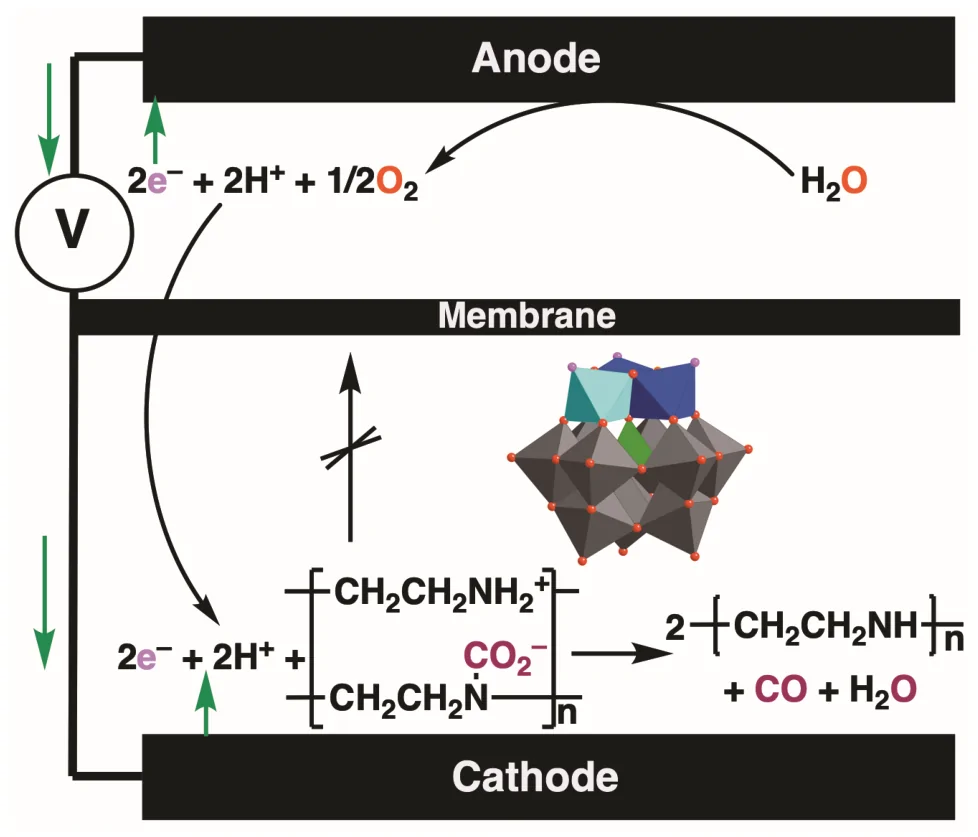
Reduction of branched polyethylenimine-derived carbamates to CO catalyzed by [SiCu_2_^II^Ga^III^(H_2_O)_3_W_9_O_37_]^9−^. W: dark grey; Cu: blue; Ga: turquoise; O: red. For clarity the branched polyethylenimine is presented as a linear polymer.

The research described herein asks several questions. First, are transition-metal-substituted polyoxometalates, previously studied for e-CO_2_RR [47], also active catalysts for e-RCC reactions and then which are the most active? Second, do small-molecule amines and their corresponding carbamates diffuse through commonly used PEMs or AEMs from the cathode to anode, and if so, what is a strategy to mitigate this diffusion? Third, are small-molecule amines and their corresponding carbamates oxidized at the anode under typical e-RCC cell potentials? As will be shown below and summarized in Figure 1, [SiCu_2_^II^Ga^III^(H_2_O)_3_W_9_O_37_]^9−^ was found to be the most active for e-RCC reactions, but carbamates based on small molecule amines quickly traverse PEMs and AEMs and are oxidized at the anode to form CO and CO_2_. To mitigate the problem of carbamate diffusion from cathode to anode cell compartments, high-molecular-weight amines were used to capture CO_2_, and the resulting carbamate solution was reduced to CO with the co-formation of H_2_ as a by-product.

## 2. Results and Discussion

**Scheme 1:**
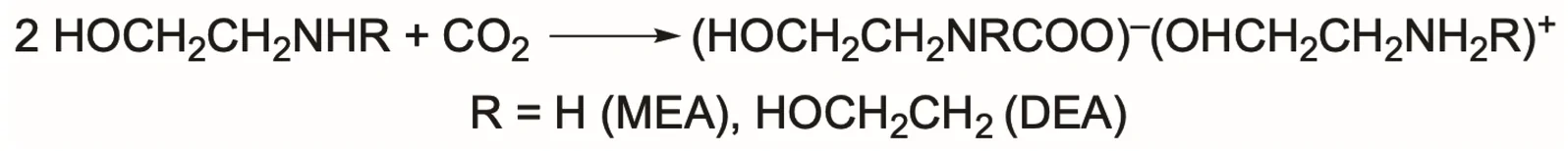
Reaction of CO_2_ with MEA and DEA.

To evaluate potential polyoxometalate compounds as catalysts for the reduction of ammonium carbamates, CO_2_ was reacted with 1:1 aqueous solutions of ethanolamine (MEA) and diethanolamine (DEA) to form the corresponding ammonium carbamates, as seen in Scheme 1.

**Figure 2:**
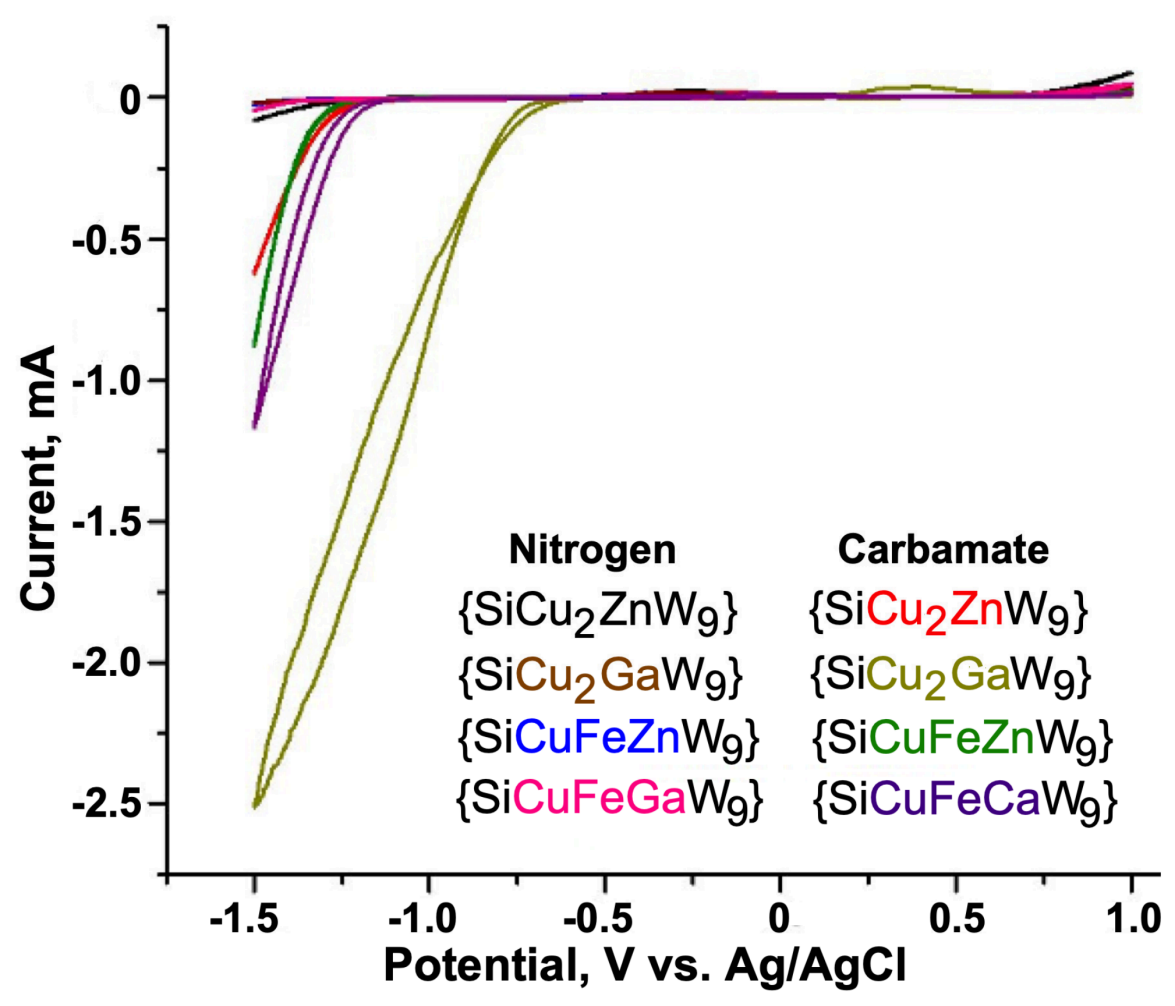
Cyclic voltammograms of four different polyoxometalates in 1:1 aqueous MEA under N_2_ and after formation of MEAC, by bubbling CO_2_ for 20 min. Conditions: 2 mM polyoxometalate, 3 mL MEA, 3 mL of DDW, glassy carbon working electrode (WE), stainless-steel wire counter electrode (CE), and Ag/AgCl reference electrode (RE), at room temperature, with scan rate 100 mV/s.

As can be seen from the ^1^H and ^13^C NMR measurements, depicted in Figures S1 and S2, CO_2_ was introduced into 1:1 by volume aqueous solutions containing either a primary amine, MEA, or a secondary amine, DEA, to afford in situ formation of aqueous ammonium carbamate species via the reaction of approximately one molar equivalent of CO_2_ with two molar equivalents of amine. After about 20 min the formation of the MEA and DEA carbamates was completed, and further addition of CO_2_ led to the decrease in pH and the formation of a small amount of bicarbonate and some dissolved CO_2_. Also, one can observe that MEA is notably more efficient in forming MEA-carbamate than DEA, and thus MEA was used to evaluate the electrocatalytic activity of polyoxometalates. Initial cyclic voltametric (CV) measurements were carried out under N_2_ and MEA carbamate (MEAC) for various polyoxometalates [SiCu_2_^II^Ga^III^(H_2_O)_3_W_9_O_37_]^9−^ {SiCu_2_GaW_9_}, [SiCu^II^Fe^III^Ga^III^(H_2_O)_3_W_9_O_37_]^8−^ {SiCuFeGaW_9_}, [SiCu_2_^II^Zn^II^(H_2_O)_3_W_9_O_37_]^10−^ {SiCu_2_ZnW_9_}, and [SiCu^II^Fe^III^Zn^II^(H_2_O)_3_W_9_O_37_]^9−^ {SiCuFeZnW_9_} chosen based on previous research that showed such polyoxometalates to be the most reactive [47], Figure 2. 

**Figure 3:**
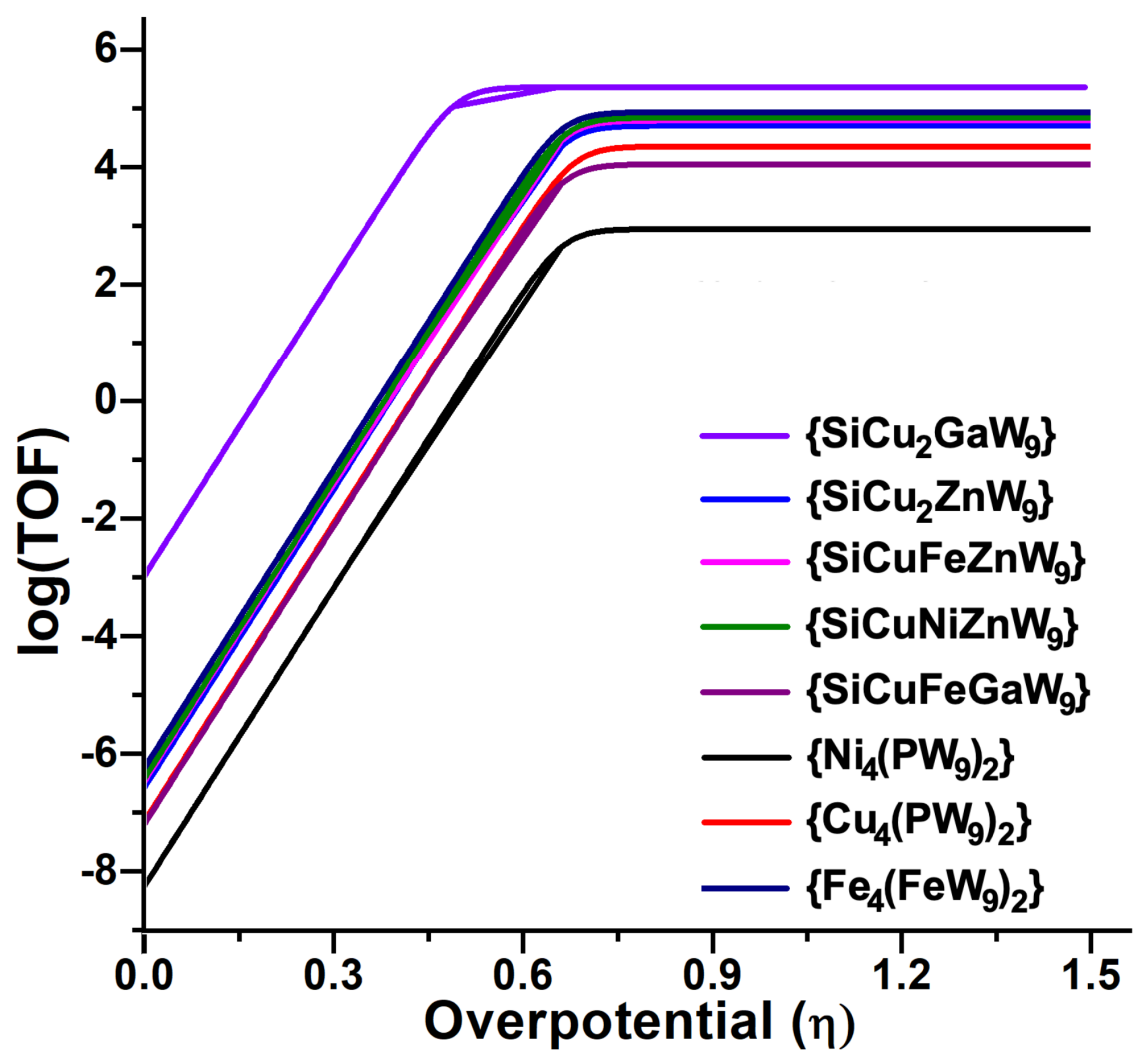
Catalytic Tafel plots for the cathodic MEAC reduction by various polyoxometalate electrocatalysts. Data was taken from cyclic voltammograms (Figure 2 and Figure S4–S7). Structures for the various complexes are presented in Figure S8.

No reduction peaks were observed of MEA-H_2_O solutions under N_2_, but after formation of MEAC, {SiCuFeGaW_9_}, {SiCu_2_ZnW_9_}, and {SiCuFeZnW_9_} and others, Figure 3 shows catalytic peaks with onset reduction potentials at −1.18 to −1.22 V. Surprisingly, {SiCu_2_GaW_9_} showed significantly lower reduction onset potentials of −0.68 V and significantly higher reduction currents. This contrasts with e-CO_2_RR catalyzed by these polyoxometalates where the highest activity was observed at similar potentials as those obtained with {SiCuFeGaW_9_} [47]. To support a postulate that the observed reactivity of {SiCu_2_GaW_9_} originated predominantly from the carbamate species rather than dissolved CO_2_ or bicarbonate, solutions were (a) treated with N_2_ following CO_2_ saturation (20 min), and the CV was subsequently recorded. There is no change in the onset potential and only a very slight decrease in the reduction potential was observed and (b) CV measurements after extending the CO_2_ bubbling time to up to 40 min with a concurrent reduction in pH (Figure S1) resulted in a progressive decrease in the catalytic peak current, as depicted in Figure S3. This sensitivity to the decreasing pH in solution is likely due to a decrease in the relative amount of carbamate on the electrode during the CV measurement because of competition with bicarbonate and/or lower reactivity of CO_2_ formed in situ. CVs as a function of scan rate are shown in Figure S3 and show slightly decreasing current with the increase in scan rate likely because of incomplete regeneration of active species after electron transfer with increasing scan rates.

One way to determine the maximum turnover frequencies, (TOF_max_), and compare the electrocatalytic activity of the various polyoxometalates is by calculating TOF_max_ and the overpotential, η, using a catalytic Tafel plot [48,49,50], as in Equation (1) and Figure 3.

(1) $$TOF=\frac{2k_{cat}}{1+\exp\left[\frac{F}{RT}\left(E_{MEAC}^{0}-E_{cat}^{0}\right)\right]\exp\left(-\frac{F}{RT}ɳ\right)}$$

The k_cat_ values were obtained from the CV measurements, as depicted in Figure 2 and Figures S4–S7, and E^0^_MEAC_ is the standard potential for the cathodic reaction, as demonstrated in Equation (2), and at pH = 10 it is calculated to be −0.91 V (see further details in the Materials and Methods Section). E^0^_cat_ is the standard potential of the catalyst under N_2_.

MEAC + 2 H^+^ + 2 e^–^ ⟶ 2 MEA + CO + H_2_O (2)

The results show that {SiCu_2_GaW_9_} has the lowest overpotential (η = 0.54 V) at the highest log TOF_max_ = 5.17 while the other catalysts have overpotentials of η = 0.69 V with a range of lower TOF_max_ values.

Initial controlled potential electrolysis (CPE) experiments were carried out in an undivided cell to sample the products of a MEAC reduction reaction. For example, a CPE reaction of a 6 mL 1:1 solution of MEA: H_2_O treated for 20 min with CO_2_ containing 2 mM {SiCu_2_GaW_9_}, at room temperature for 15 h at −1.5 V versus Ag/AgCl using a glassy carbon (d = 3 mm) WE and a stainless-steel CE, yielded 25 µmol CO and 25 µmol H_2_ as only products. Analysis was by gas chromatography with a thermal conductivity detector for the gas phase (GC-TCD) and ^1^H NMR for the liquid phase. To verify that the CO formed was indeed from the in situ prepared MEAC, an identical experiment was performed where MEAC was prepared using ^13^CO_2_. Analysis by GC with a mass selective detector (MSD) revealed the formation of >90% unlabeled ^12^CO_2_ indicating an anodic oxidation of MEA and/or MEAC. To further understand this anodic oxidation, (1) the CV for the oxidation of MEA, DEA, diethylamine and their corresponding carbamates were tested, and (2) the transport of MEAC was conducted through a Nafion membrane often used in divided cell configurations. One can see in Figure S9 that the amines themselves are quite stable to oxidation up to 1.5 V versus Ag/AgCl; however, the corresponding carbamates are oxidized at this potential; diethylamine carbamate > DEAC > MEAC. Furthermore, the transport of MEAC through a Nafion membrane in a H-cell configuration was measured by ^1^H NMR spectra as a function of time under open circuit conditions starting with MEAC in the cathode cell only, as depicted in Figure S10. As can be seen MEAC easily traverses the Nafion membrane within a short period of time. It should also be noted that upon crossing the Nafion membrane, MEAC was quantitatively hydrolyzed to MEA. Finally, reduction of MEAC was carried out in both an H-cell and a membrane electrode assembly (electrolyzer). The anode used for H_2_O oxidation was IrO_2_ on platinized Ti felt. The cathode was a polyoxometalate catalyst supported on a porous carbon gas diffusion electrode, and Sustainion^®^ X37-50 grade 60 and Pipirion^®^ were used as AEMs to prevent MEAC decomposition. The headspace above the cathode and anode cells was analyzed separately by GC-TCD. The results showed that CO was found almost exclusively in the anode cell, supporting the above-mentioned indications that CO can be formed from MEAC oxidation rather than MEAC reduction. H_2_ was formed exclusively at the cathode. See the Supplementary Materials for more details, specifically Figure S11.

Given that the catalytic reduction of MEAC as observed by CV could not be translated to a divided cell configuration because of fast transport of such small-molecule-derived carbamates through commercial PEMs and AEMs, the solution to this problem would be to use high-molecular-weight polyamines as CO_2_-capturing agents that would form carbamate units on the polymer chain and would not traverse PEMs or AEMs. Common and commercial branched polyethylenimine polymers (PEIs) that contain ~25% tertiary, ~50% secondary, and ~25% primary amines, already known as carbon-capture compounds [51,52,53,54], and available in a range of molecular weights from 800 to 1,000,000, were used for the e-RCC reaction in a membrane electrode assembly electrolyzer. The amount of CO_2_ bound by treatment of 1:1 PEI:H_2_O was ~80 mg CO_2_/g 25 K PEI (7.8 mol%/amine unit) and ~90 mg CO_2_/g 1 M PEI (8.9 mol%/amine unit), similar to previous reports [52,53].

**Figure 4:**
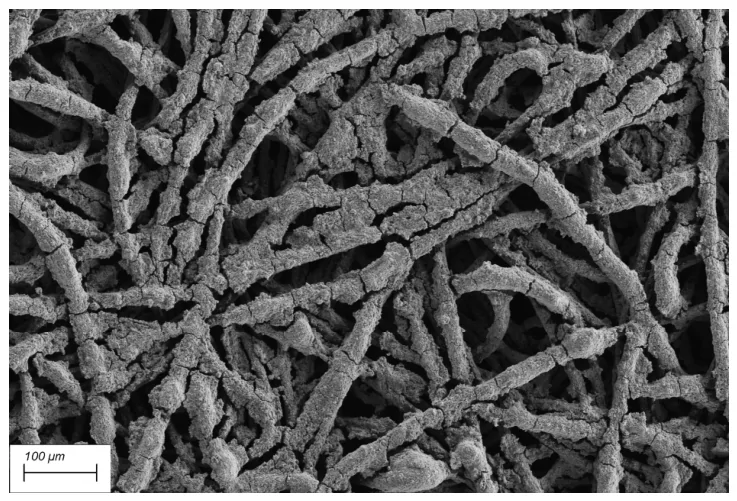
HRSEM image of {SiCu_2_GaW_9_} on a carbon GDL using an Aemion^®^ ionomer-based ink.

In the first step using the polyanionic properties of the polyoxometalate compounds and the polycationic properties of typical anion exchange ionomers based on quaternary ammonium sites, polyoxometalates were heterogenized onto porous carbon supports as gas diffusion layers (GDLs). The GDL electrode was fabricated by preparing an ink containing 80 wt% polyoxometalate, 10 wt% Aemion^®^ ionomer, and 10 wt% carbon black, dispersed in a mixture of isopropanol and water. The suspension was homogenized by ultrasonication for 30 min and subsequently spray-coated onto a carbon paper substrate (Freudenberg H23C6 GDL with a microporous layer and hydrophobic surface treatment) using an air gun operated at 2 bar pressure. The catalyst morphology was evaluated on a high-resolution scanning electron microscope (HRSEM). The HRSEM images at 500× magnitude showed a very good dispersion of {SiCu_2_GaW_9_} on the GDL, as depicted in Figure 4 in comparison to the untreated carbon paper GDL in Figure S12. EDS measurements showed homogeneous coverage of {SiCu_2_GaW_9_} on the GDL as would be expected for a molecular compound, as depicted in Figure S13.

Initial exploratory CPE reactions were carried out in an cell using {SiCu_2_GaW_9_}, {SiCuFeGaW_9_}, or {Ni_4_(PW_9_)_2_} on a 1 cm^2^ carbon paper GDL as described above and pictured in Figure 4 as WE, a IrO_2_ on Pt-Ti felt separated by a glass frit as CE, and a Ag/AgCl RE. CPE was carried out in a 18 mL glass vial containing 1 mL of PEI (molecular weight 1 million), 3 mL of DDW, 2 mL of Na_7_DTPMP (heptasodium diethylenetriaminepentamethylenephosphonate 25% (*w*/*v*) in H_2_O), and 0.8 M Na_2_SO_4_·10H_2_O under 1 bar CO_2_ reacted at −1.5 V, −1.3 V, and −1.0 V versus Ag/AgCl for 15 h at room temperature. CO and H_2_ were the only observed products. Although the separation of the in situ formed carbamate of PEI from the anode was only by a frit, the importance of this measurement lies in the observation that {SiCu_2_GaW_9_} was active down to −1 V, while {SiCuFeGaW_9_} and {Ni_4_(PW_9_)_2_} were active at only more negative potentials, with {Ni_4_(PW_9_)_2_} as the poorest catalyst, as depicted in Figure S14. This supports the finding that {SiCu_2_Ga W_9_} is uniquely active at lower overpotentials as observed in the CV measurements. A control experiment to verify the source of CO from CO_2_ using PEI-carbamate prepared with ^13^CO_2_ showed formation of ^13^CO only.

**Figure 5:**
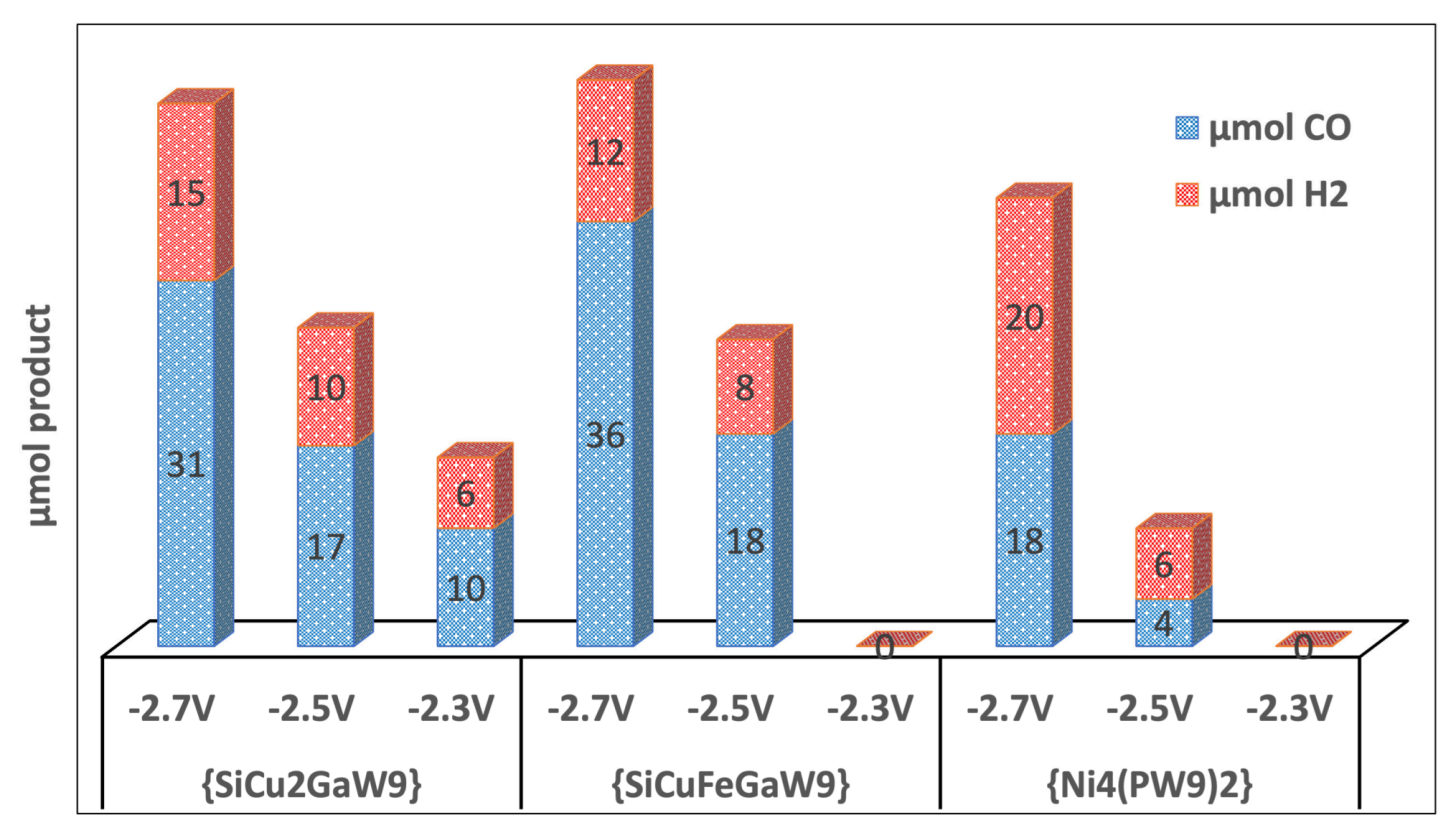
Controlled-voltage electrolysis comparing {SiCu_2_GaW_9_}, {SiCuFeGaW_9_}, or {Ni_4_(PW_9_)_2_} in an electrolyzer. Cathode: 1 cm^2^ carbon paper GDL from ink containing 10 µmol polyoxometalate, 2 mg Aemion^®^ ionomer, and 5 mg carbon black. Anode: IrO_2_ on Pt-Ti felt as counter electrode. Catholyte: 1 mL PEI (molecular weight 1 M), 3 mL H_2_O, 2 mL Na_7_DTPMP 25% (*w*/*v*) in H_2_O, and 0.8 M Na_2_SO_4_·10H_2_O treated for 20 min under a flow of CO_2_. Anolyte was Na_7_DTPMP 25% (*w*/*v*) in H_2_O and 0.8 M Na_2_SO_4_·10H_2_O. Nafion 107 membrane, room temperature, and time of 15 h. Reactions were carried out in duplicate with minimal differences. Total faradaic efficiencies were moderate and typically less than 40%; see Table S1 for detailed data.

To solidify the advantage of the use of PEI for e-RCC, CPE was carried out in a membrane electrode assembly electrolyzer. Controlled-voltage electrolysis (CVE) was carried out in 4 cm^2^ electrolyzer using {SiCu_2_GaW_9_}, {SiCuFeGaW_9_}, or {Ni_4_(PW_9_)_2_} on a GDL cathode as described above (1 cm^2^ area) as WE. The catholyte was prepared from 1 mL PEI and 3 mL H_2_O, 2 mL Na_7_DTPMP 25% (*w*/*v*) in H_2_O, and 0.8 M Na_2_SO_4_·10H_2_O. The catholyte was treated for 20 min under a flow of CO_2_ gas to yield the corresponding PEI-carbamate and then purged with N_2_ and kept under 2 bar N_2_. The anolyte was Na_7_DTPMP 25% (*w*/*v*) in H_2_O and 0.8 M Na_2_SO_4_·10H_2_O. The anolyte and catholyte solutions were separated by a Nafion 107 membrane. CVE was carried out at 2.7, 2.5, and 2.3 V for 15 h at room temperature. Analysis of the gas phase by GC-TCD showed that CO and H_2_ were the only gaseous products formed while analysis by ^1^H NMR of the liquid phase from reactions carried out in D_2_O showed no discernible formation of any soluble products. The results are summarized in Figure 5.

Post-reaction analysis by NMR showed that no polyethyleneimine or corresponding carbamate had diffused into the anolyte solution, as depicted in Figure S15. The {SiCu_2_GaW_9_} catalyst appeared to be stable after 15 h under CVE conditions, as depicted in Figure S16. There was no evidence of dissolution of the polyoxometalate or component elements as measured by ICP-MS. There were only statistically insignificant (±5%) amounts of Cu, Fe, Ga, or Ni above the background amounts of the elements measured in solution after the reactions. The results indeed confirm that {SiCu_2_GaW_9_} can yield product at the lowest overpotential; although, at more negative potentials of −2.7 V, {SiCuFeGaW_9_} yielded similar results. For both polyoxometalates the CO:H_2_ ratios hovered around 2:1. On the other hand, {Ni_4_(PW_9_)_2_} was less reactive and yielded H_2_ in preference to CO. A comparison of the use of different molecular weight PEI, 1,000,000 versus 25,000, showed that the former yields better results, as depicted in Figure S17. Tabulated results including the FE (%), charge density (J, mA/cm^2^), and total charge (Q, C) can be found in Table S1. Using the conditions described in Figure 5, control experiments (1) without catalyst (just GDL) showed no product formation; (2) PEI not treated with CO_2_ also showed no product formation; (3) experiments with CO_2_ dissolved in water yield only small trace amounts of CO; and (4) an experiment with ^13^CO_2_ showed only the formation of ^13^CO.

## 3. Materials and Methods

**Polyoxometalate Synthesis**. The alkali metal salts of {SiCu_2_ZnW_9_}, {SiCu_2_GaW_9_}, {SiCuFeGaW_9_}, {SiCuFeZnW_9_}, and {SiCuNiZnW_9_} were synthesized and analyzed as previously reported [47]. Similarly, the alkali metal salts of {Ni_4_(PW_9_)_2_}, {Cu_4_(PW_9_)_2_}, and {Fe_4_(FeW_9_)_2_} were also prepared by procedures reported in the literature [55,56,57].

**Electrochemistry.** Electrochemical experiments were carried out using a Biologic, Seyssinet-Pariset, France multichannel VSP 201 potentiostat. Cyclic voltammograms of 2 mM polyoxometalate in 3 mL water and 3 mL MEA were measured at different scan rates under 1 bar N and after formation of MEAC by treatment with a flow of 1 bar CO_2_ (99.99%) for 20 min. Glassy carbon (d = 3 mm) WE, a stainless-steel wire CE, and Ag/AgCl (3M KCl) RE. Similar CV measurement to evaluate the oxidation of MEA and MEAC were carried out in the same manner by exchanging the WE and CE. To extract catalytic parameters from the CV measurements, we used the equation proposed by Kubiak and coworkers for deriving the catalytic rate constant, k_cat_ [48], namely Equation (1), where R is the universal gas constant, T = temperature, F = Faraday constant, ʋ = scan rate, n is the number of electron-transfer processes that occur at the electrode per catalyst and equals 2; n′ is the number of catalysts equivalents required per catalysts and equals 1; i_cat_ is the current under CO_2_; i_p_ is the current under N_2_; and k_cat_ is the catalytic rate constant.

(3) $$\frac{i_{cat}}{i_{p}}=\frac{1}{0.446}\sqrt{\frac{RT}{nFʋ}n^{\prime }k_{cat}}\;$$

**Figure 6:**
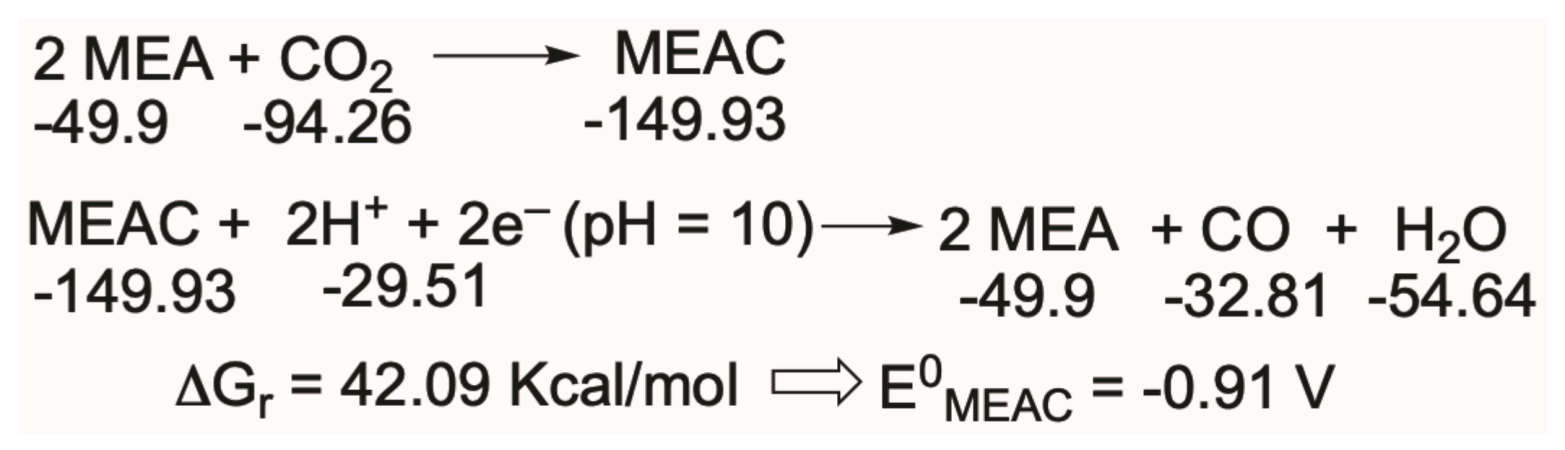
Calculation of E^0^_MEAC_ from literature ΔG_f_ values of ΔG_f_ for MEA, CO_2_, CO, and H_2_O were taken directly from the literature [58,59]. The ΔG_f_ for 2 H^+^ + 2 e^–^ was calculated from E = 0.0591 V × pH. The ΔG_f_ of MEAC was deduced from DFT-calculated literature values [60].

The interdependency of turnover frequency (TOF) and overpotential (η) are based on the intrinsic properties of the catalyst and can be calculated from the difference between the applied potential and the standard potential for the reduction of CO_2_ to CO, independent of contingent factors such as the cell characteristics. The relevant catalytic Tafel plots were calculated using Equation (3) [49,50]. E^0^_MEAC_, the standard potential for the reduction of carbamate to CO under the specific conditions of catalysis, was calculated using literature data for ΔG_f_ values and is shown in Figure 6; E^0^_MEAC_ = −0.91 V. The pH = 10 value was the one measured before the CV measurements (Figure S1). The presence of bicarbonate or dissolved CO_2_ was not observed and therefore not considered.

**Controlled-voltage electrolysis.** Reactions in 4 cm^2^ electrolyzer (with 1 cm^2^ active area). Cathode: a carbon-paper GDL electrode was fabricated by preparing an ink containing 80 wt% polyoxometalate cesium salts of ({SiCu_2_GaW_9_}, {SiCuFeGaW_9_}, or {Ni_4_(PW_9_)_2_}), 10 wt% Aemion ionomer, and 10 wt% carbon black, dispersed in a mixture of isopropanol and water. The suspension was homogenized by ultrasonication for 30 min and subsequently spray-coated onto a carbon-paper substrate (Freudenberg H23C6 GDL with a microporous layer and hydrophobic surface treatment) using an air gun operated at 2 bar pressure. Catholyte: 1 mL PEI, 3 mL H_2_O, 2 mL Na_7_DTPMP 25% (*w*/*v*) in H_2_O, and 0.8 M Na_2_SO_4_·10H_2_O treated for 20 min under a flow of CO_2_. Anode: IrO_2_/Pt-Ti felt. Anolyte: 3 mL DDW, 2 mL Na_7_DTPMP (25% *w*/*v*), and 0.8 M sodium sulfate decahydrate. Reactions were carried out under 2.5 bar N_2_ for 15 h at room temperature at cell voltages ranging from 2.3 to 2.7 V, respectively, with an anodic potential of ∼1.0 V versus SHE. The analysis of the products at the gas phase was conducted by using a HP 6890 GC-TCD, Santa Clara, CA, USA using a ShinCarbon ST 80/100 micropacked 2 m × 0.53 mm ID column with He as a carrier gas. Hydrogen was analyzed separately using a GOWMAC GC-TCD, Bethlehem, PA, USA, configured with two columns in series (4′ by 1/8” Hayesep T; 10′ by 1/8” Molecular sieve 5A) with Ar as a carrier gas carrier. CO and H_2_ were observed as the only products. Experiments using ^13^CO_2_-labeled carbamates were analyzed by GC-MS using a molecular sieve PLOT column (30 m × 0.32 mm)

**^1^H NMR** spectra were recorded on a Bruker Avance III 300 MHz spectrometer, Ettlingen, Rheinstetten, Germany at 298 K and referenced to the solvent shift. Data manipulations were completed using MestReNova 14.3.2 and TopSpin3.7 software.

**HRSEM** imaging was carried out on a Zeiss MERLIN high-resolution scanning electron microscope, Oberkochen, Germany containing an Inlens detector for higher energy secondary electrons for clear topographic mapping.

## 4. Conclusions

Others have noted an e-RCC reaction of captured carbamate with small-molecule amines such as MEA to form MEAC as substrate for the formation of CO to indirectly reduce low atmospheric concentrations of CO_2_. We have found, however, that such carbamates easily diffuse through membranes in divided cell configurations, e.g., H-cells or membrane assembly electrolyzers, and are oxidized at the anode. To circumvent this problem, we have shown that high-molecular-weight PEI can be used as a proven CO_2_ capturing agent. The corresponding carbamates can yield CO along with H_2_ in ~2:1 ratio in a CVE reaction using a membrane electrode assembly electrolyzer with a supported {SiCu_2_GaW_9_} catalyst. The experiments demonstrated that {SiCu_2_GaW_9_} reduces polyammonium carbamates at a significantly less negative potential (–1.0 V vs. Ag/AgCl), compared to other tested polyoxometalate catalysts, and produces CO with H_2_ as a co-product. Typically, current densities of 1–2 mA/cm^2^ were obtained with only moderate faradaic efficiencies. It is not yet possible to benchmark these results since previous e-RCC research using amine capture has not considered anodic oxidation of the small-molecule carbamates used as substrates, typically MEAC. Considering the too-low faradaic efficiencies and current densities, and limited actual turnover numbers, much more research is still required to optimize such polyamine-based e-RCC reactions.

## Data Availability

Supporting reported results can be found in the Supplementary Materials.

## Supplementary Materials

The following supporting information can be downloaded at https://www.mdpi.com/article/10.3390/molecules31183276/s1: Figure S1: ^1^H NMR spectra of 1:1 vol MEA:H_2_O and 1:1 vol DEA:H_2_O upon addition of CO_2_; Figure S2: ^13^C NMR spectra of 1:1 vol MEA:H_2_O upon addition of ^13^CO_2_; Figure S3: Cyclic voltammograms of {SiCu_2_GaW_9_}, {SiCuFeGaW_9_}, {SiCu_2_ZnW_9_}, and {SiCuFeZnW_9_} at different scan rates; Figure S4: Cyclic voltammogram of 2 mM {SiCuNiZnW_9_} under 1 bar N_2_ and MEAC; Figure S5: Cyclic voltammogram of 2 mM {Cu_4_(PW_9_)_2_} under 1 bar N_2_ and MEAC; Figure S6: Cyclic voltammogram of 2 mM {Ni_4_(PW_9_)_2_} under 1 bar N_2_ and MEAC; Figure S7: Cyclic voltammogram of 2 mM {Fe_4_(FeW_9_)_2_} under 1 bar N_2_ and MEAC; Figure S8: Polyhedral presentation of polyoxometalates tested; Figure S9: Anodic cyclic voltammetry of MEA, DEA, and Diethylamine and their corresponding carbamates; Figure S10: Transport of MEAC through a Nafion membrane as measured by ^1^H NMR; Figure S11: Controlled potential electrolysis of MEAC in divided cells; Figure S12: HRSEM image of the carbon GDL; Figure S13: Elemental mapping (HR-SEM; EDS) of {SiCu_2_GaW_9_} on a carbon GDL; Figure S14: Controlled potential electrolysis comparing {SiW_9_Cu_2_Ga}, {SiW_9_CuFeGa}, or {Ni_4_(PW_9_)_2_}; Figure S15: Post-reaction analysis of catholyte and anolyte ^1^H NMR; Figure S16: IR spectra of {SiCu_2_GaW_9_} after controlled-voltage electrolysis; Figure S17: Controlled-voltage electrolysis comparing PEI (1 M) and PEI (25 K) in an electrolyzer; Table S1: Faradaic efficiencies, current densities, product distributions, and charge using three catalysts, two polyethylenimines, and at three cell voltages.

## Abbreviations

AEM	Anion exchange membrane
CE	Counter electrode
CPE	Controlled-potential electrolysis
CV	Cyclic voltammetry
DEA	Diethanolamine
CCU	Carbon capture and utilization
DDW	Double-distilled water
e-CO_2_RR	Electrochemical CO_2_ reduction reaction
e-RCC	Electrochemically reactive capture
GC-TCD	Gas chromatography–thermal conductivity detector
GDL	Gas diffusion layer
HRSEM	High-resolution scanning electron microscopy
MEAC	Carbamate of monoethanolamine
Na_7_DTPMP	Heptasodium diethylenetriaminepentamethylenephosphonate
PEI	Polyethylenimine
PEM	Proton exchange membrane
RE	Reference electrode
TOF	Turnover frequency (1/s)
WE	Working electrode
