# Supplementary material for: Electrochemical Reduction of Amine-Captured Carbon Dioxide Catalyzed by Transition-Metal Substituted Polyoxometalates
Source: Molecules. 2026 Sep 16;31(18):3276. doi: 10.3390/molecules31183276 (PMC13609264; doi:10.3390/molecules31183276)
Supplement: Supplementary file 1 [file molecules-31-03276-s001.zip › molecules-4502053-supplementary.pdf]

Electrochemical Reduction of Amine Captured Carbon Dioxide Catalyzed by Transition Metal Substituted Polyoxometalates

Dima Azaiza-Dabbah and Ronny Neumann

Supplementary Materials

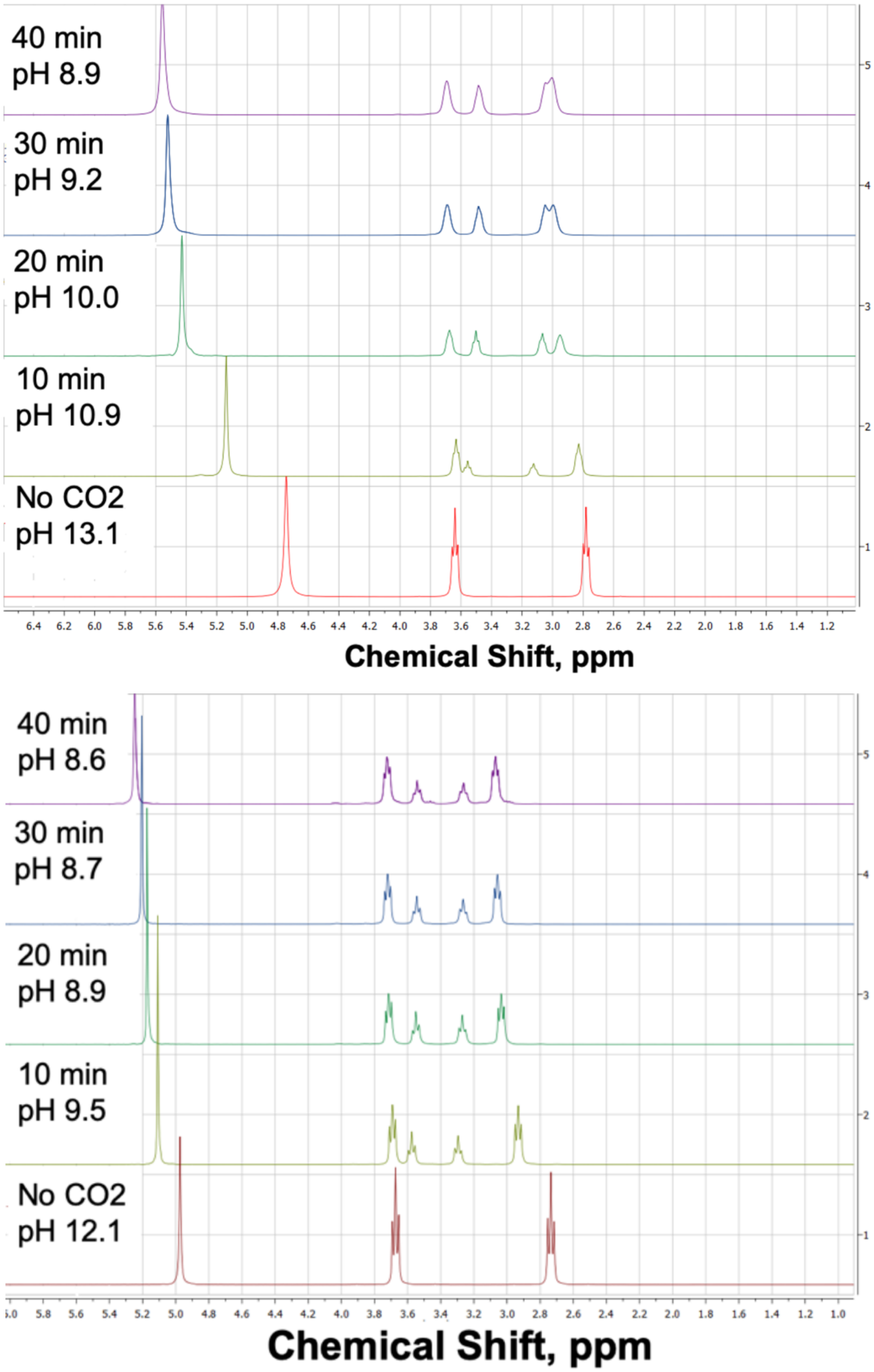

Figure S1. ^{1}H NMR spectra of 1:1 vol MEA:H_2O (top) and 1:1 vol DEA:H_2O (bottom) upon addition of CO_2 by bubbling for different time periods.

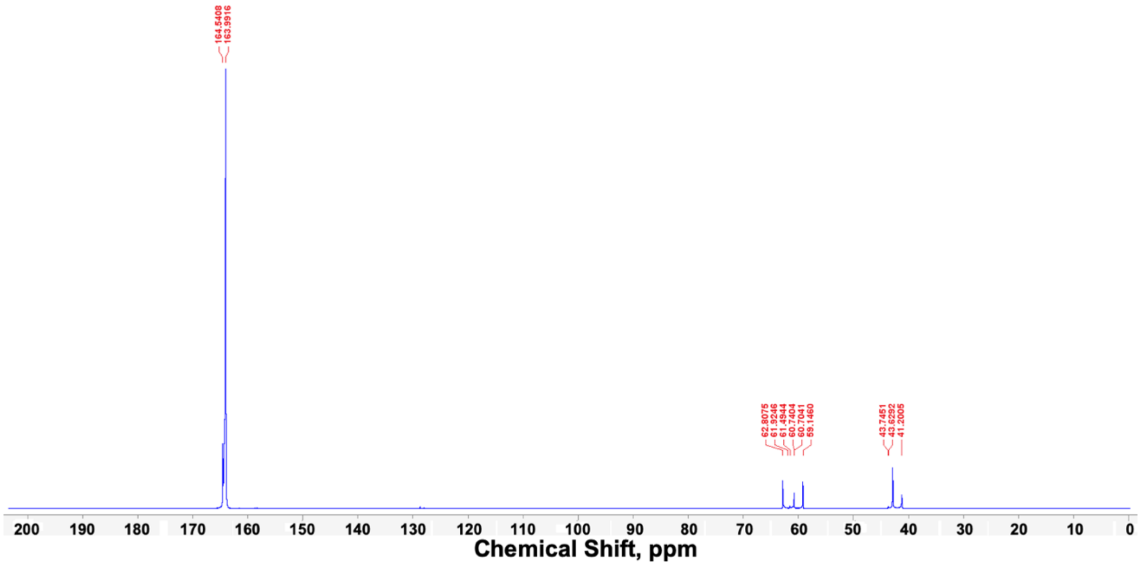

Figure S2. ^{13}C NMR spectra of 1:1 vol MEA:H_2O upon addition of $^{13}CO_2$ by bubbling for 20 min

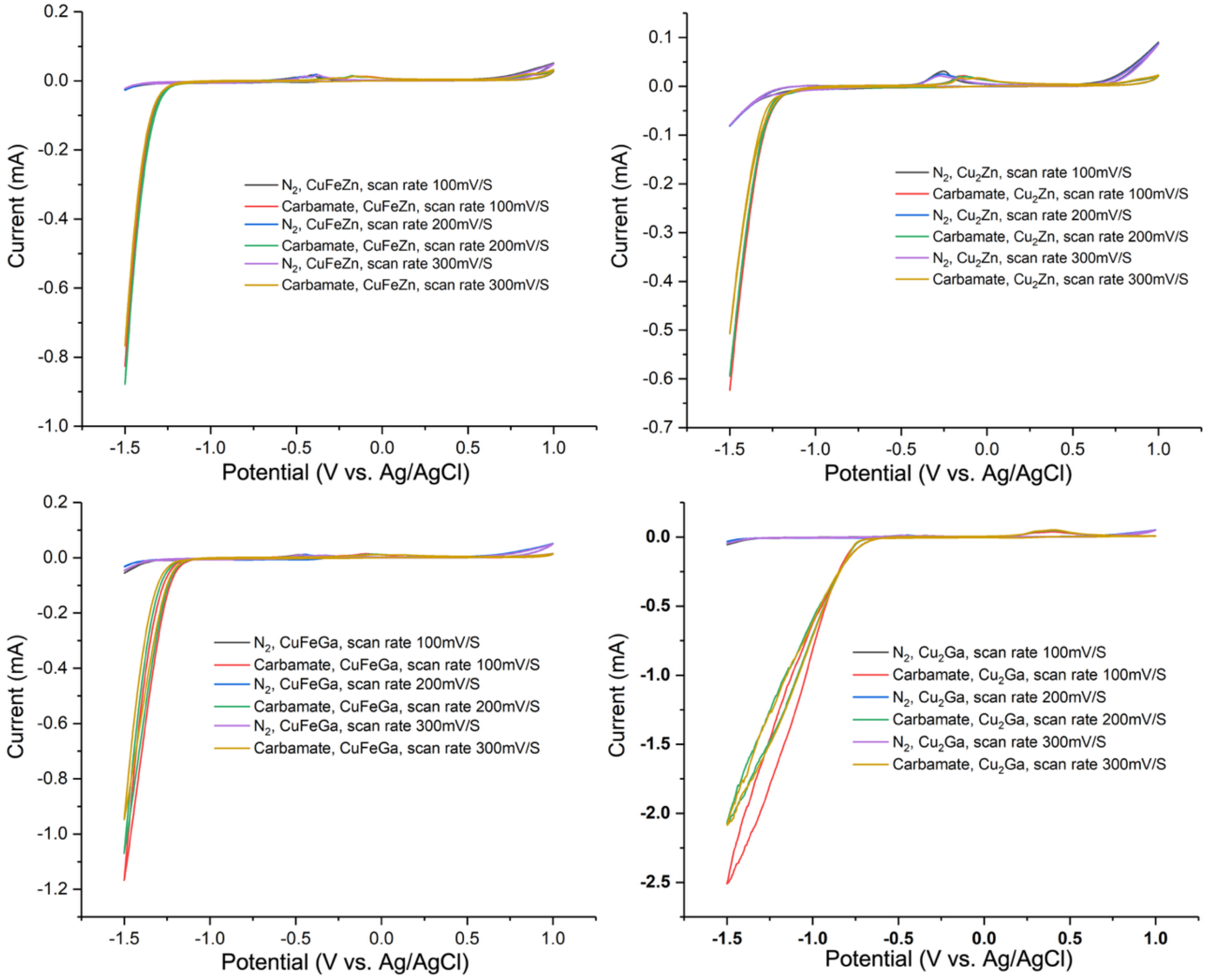

Figure S3. Cyclic voltammograms of {$SiCu_2GaW_9$}, {$SiCuFeGaW_9$}, {$SiCu_2ZnW_9$}, and {$SiCuFeZnW_9$} at different scan rates in 1:1 aqueous MEA under N_2 and after formation of MEAC, by bubbling CO_2 for 20 min. Conditions: 2 mM polyoxometalate, 3 mL MEA, 3 mL of DDW, WE-glassy carbon, CE-stainless steel wire and RE-Ag/AgCl at room temperature, scan rates 100 mV/s, 200 mV/s, 300Mv/s.

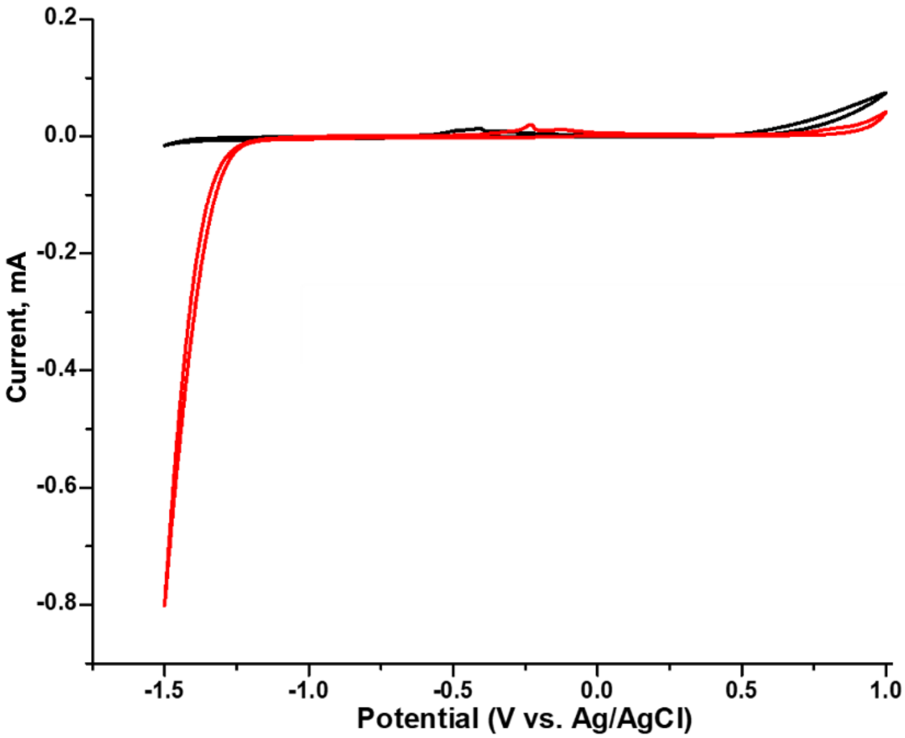

Figure S4. Cyclic voltammogram of 2mM {SiCuNiZnW$_9$} of under 1 bar N_2 (black) and MEAC (red) from MEA:DDW (1:1). Glassy carbon working electrode, stainless steel wire counter electrode and Ag/AgCl reference electrode. scan rate 100 mV/s.

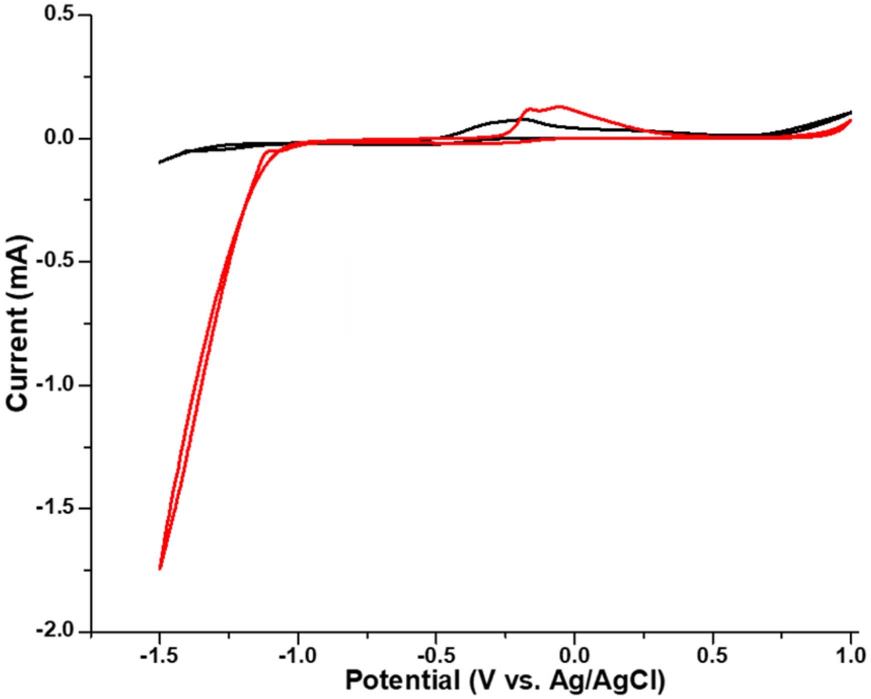

Figure S5. Cyclic voltammogram of 2mM {$Cu_4(PW_9)_2$} of under 1 bar N_2 (black) and MEAC (red) from MEA:DDW (1:1). Glassy carbon working electrode, stainless steel wire counter electrode and Ag/AgCl reference electrode. scan rate 100 mV/s.

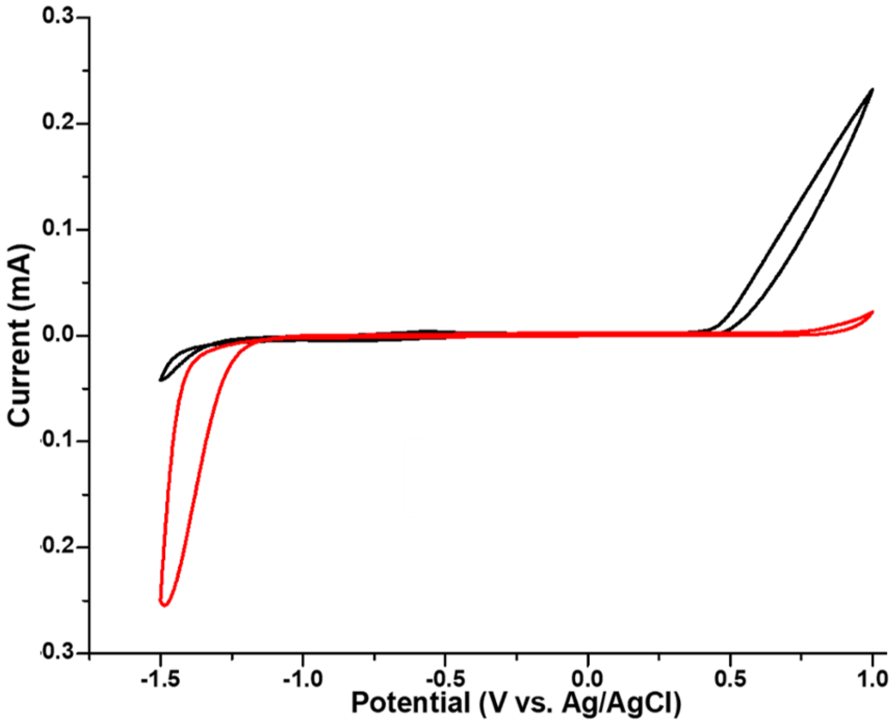

Figure S6. Cyclic voltammogram of 2mM $\{Ni_4(PW_9)_2\}$ of under 1 bar N_2 (black) and MEAC (red) from MEA:DDW (1:1). Glassy carbon working electrode, stainless steel wire counter electrode and Ag/AgCl reference electrode. scan rate 100 mV/s.

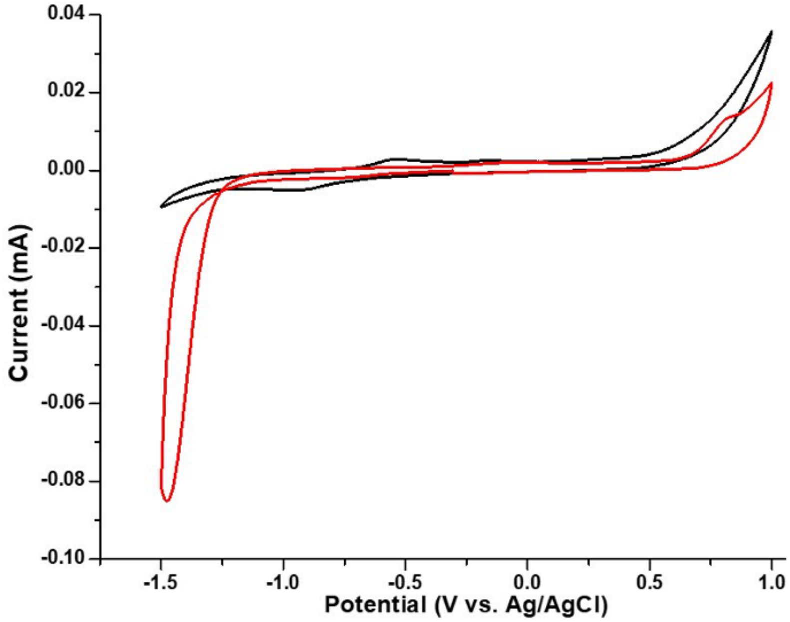

Figure S7. Cyclic voltammogram of 2mM $\{Fe_4(FeW_9)_2\}$ of under 1 bar N_2 (black) and MEAC (red) from MEA:DDW (1:1). Glassy carbon working electrode, stainless steel wire counter electrode and Ag/AgCl reference electrode. scan rate 100 mV/s.

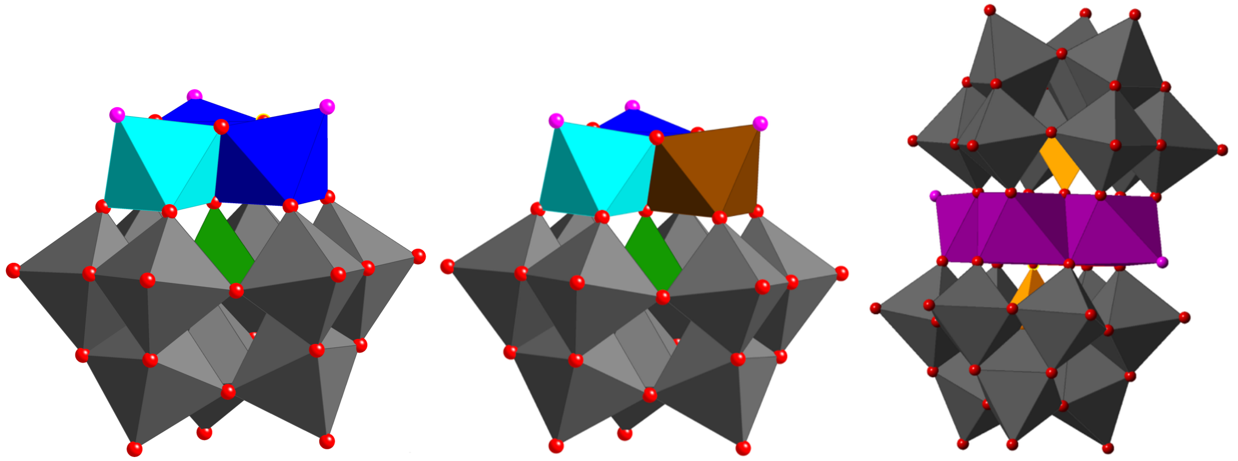

Figure S8. Polyhedral presentation of polyoxometalates tested. Left: {$SiCu_2ZnW_9$} and {$SiCu_2GaW_9$}; Cu – blue; Zn/Ga – turquoise; Si – green, W – grey, O- red. Middle: {$SiCuFeGaW_9$}, {$SiCuFeZnW_9$} an {$SiCuNiZnW_9$}; Cu – blue; Zn/Ga – turquoise; Fe/Ni – brown; Si – green, W – grey, O- red. Right: Cu/Fe/Ni – magenta; P – orange, W – grey, O- red.

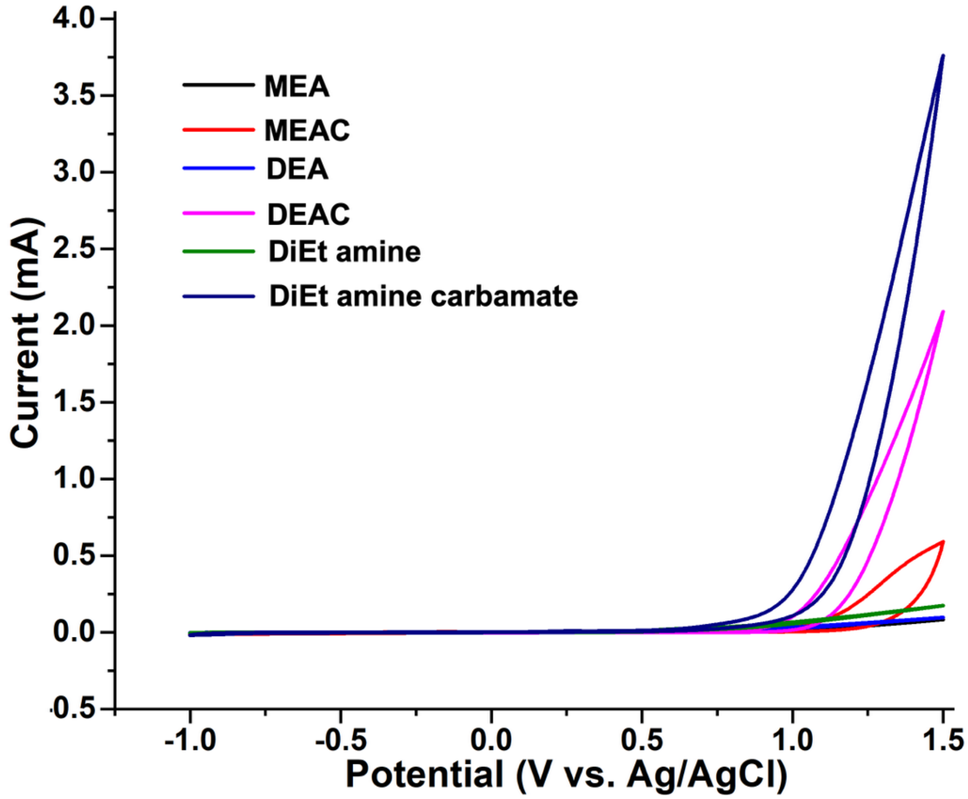

Figure S9. Anodic cyclic voltammetry of MEA, DEA and diethylamine and their corresponding carbamates. Conditions: 3 mL amine and 3 mL DDW under nitrogen and the same solutions after treatment with CO_2 for 20 min. Glassy carbon working electrode, stainless-steel wire counter electrode, and Ag/AgCl reference electrode, room temperature. Scan rate 100 mV/s

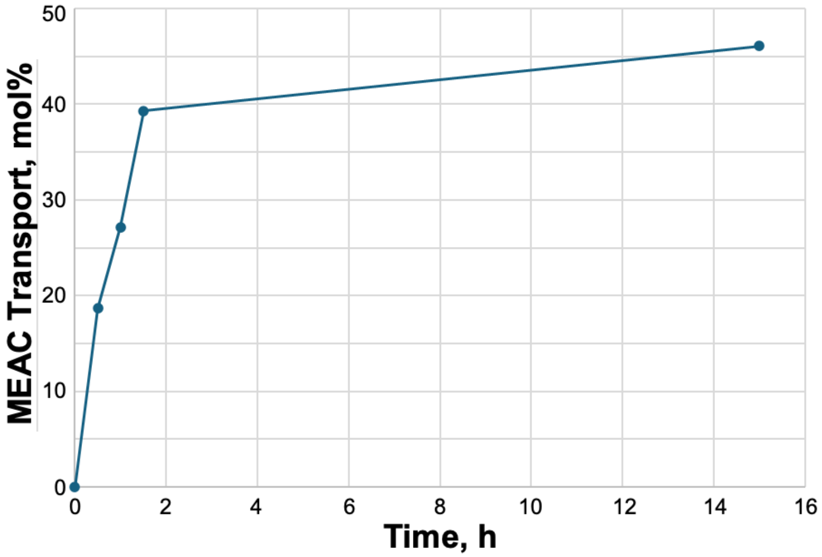

Figure S10. Transport of MEAC through a Nafion membrane as measured by ^{1}H NMR

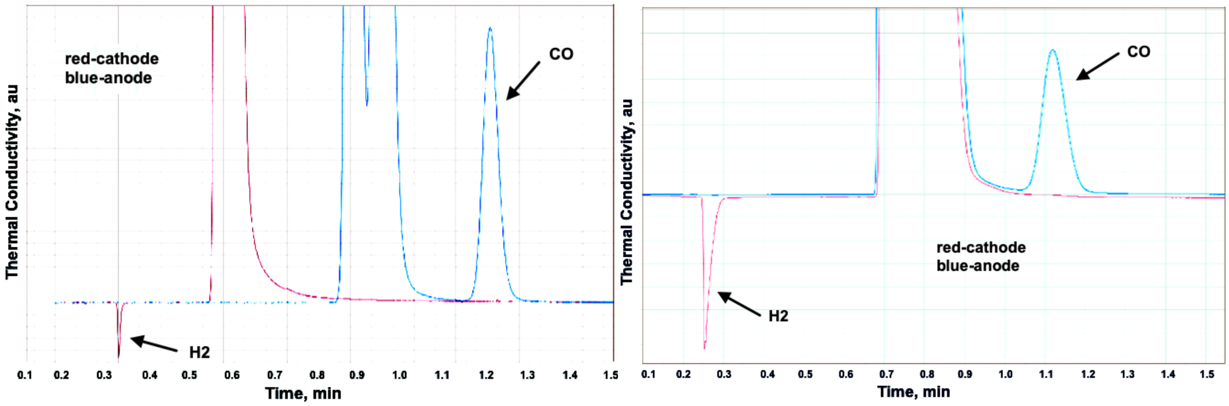

Figure S11. Controlled potential electrolysis of MEAC in divided cells. Left: Membrane electrode assembly (electrolyzer) at 3.8 V. Right: H-cell at 1.5 V versus Ag/AgCl. Anode: IrO_2 on Pt/Ti fiber felt; 1 M $KHCO_3$. Cathode: {SiCuFeGaW9} supported on a Freudenberg H23 carbon GDL: 1:1 MEA:H_2O treated for 20 min with a flow of CO_2. Membrane: Sustanion 37X. Gas phase analyzed separately at cathode and anode using an Agilent 8890 GC chromatograph with two TCD detectors and using two ShinCarbon ST 80/100 Restek columns. He was the eluent. Note that the peak areas are not linear to amount – the thermal conductivity of H2 is similar to that of He thus the H_2 peaks are small (~10X) relative to actual amount.

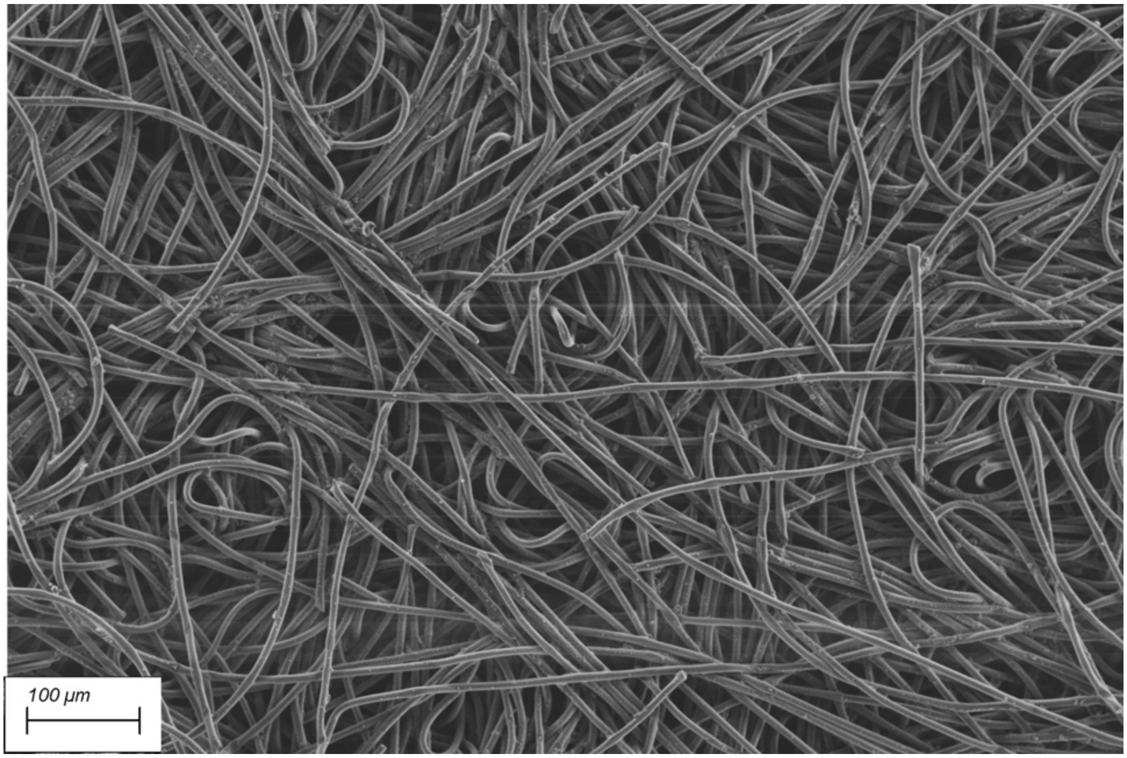

Figure S12. HRSEM image of the carbon GDL (Freudenberg H23C6 GDL with a microporous layer and hydrophobic surface treatment).

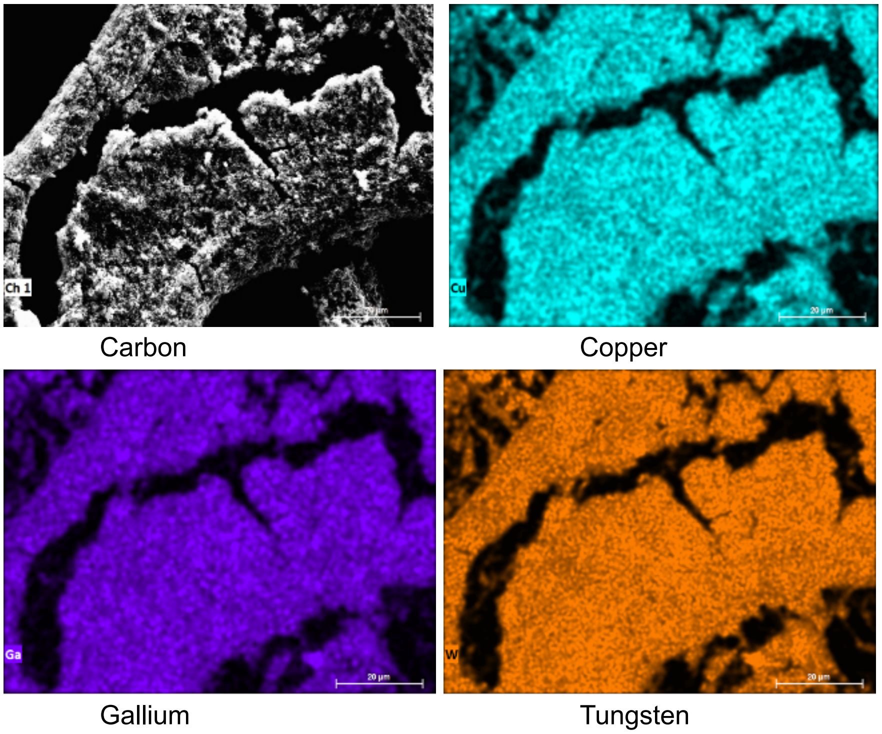

Figure S13. Elemental mapping (HR-SEM, EDS) of $\{SiCu_2GaW_9\}$ on a carbon GDL using an Aemion® ionomer based ink showing uniform distribution of the active metals (Cu, Ga, W) on the carbon substrate.

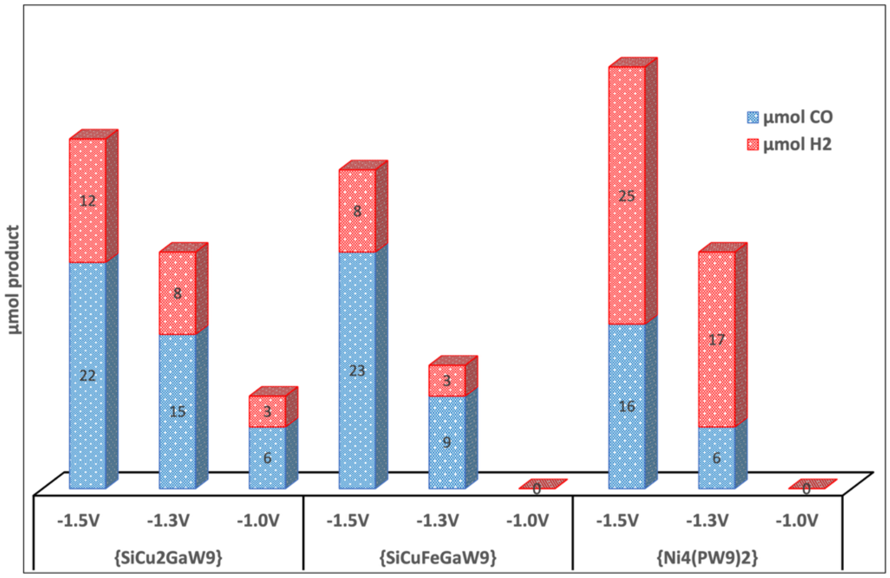

Figure S14. Controlled potential electrolysis comparing {SiW_9Cu_2Ga}, {$SiW_9CuFeGa$}, or {$Ni_4(PW_9)_2$} in a cell where the anode is separated from the cathode by a frit. Cathode: 1 cm^2 carbon paper GDL fabricated by preparing an ink containing 80 wt% polyoxometalate, 10 wt% Aemion ionomer and 10 wt% carbon black, dispersed in a mixture of isopropanol and water. The suspension was homogenized by ultrasonication for 30 min and subsequently spray-coated onto a carbon paper substrate (Freudenberg H23C6 GDL with a microporous layer and hydrophobic surface treatment) using an air gun operated at 2 bar pressure. Anode: IrO_2 on Pt-Ti felt separated by a glass frit as counter electrode. Ag/AgCl reference electrode. CPE was carried out in a 18 mL glass vial containing 1 mL of PEI (molecular weight 1 million), 3 mL of DDW, 2 mL of Na_7DTPMP (heptasodium diethylenetriaminepentamethylenephosphonate 25% (w/v) in H_2O) and sodium sulfate decahydrate (0.8 M) under 1 bar CO_2 reacted at -1.5 V, -1.3 V and -1 V versus Ag/AgCl for 15 h at room temperature. CO and H_2 were the only observed products.

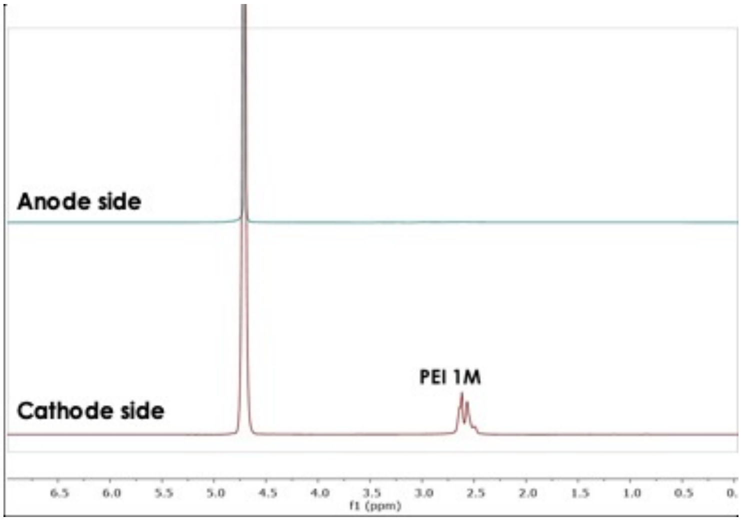

Figure S15. Post-reaction analysis of catholyte and anolyte ^{1}H NMR showing no diffusion of PEI during the course or the CVE.

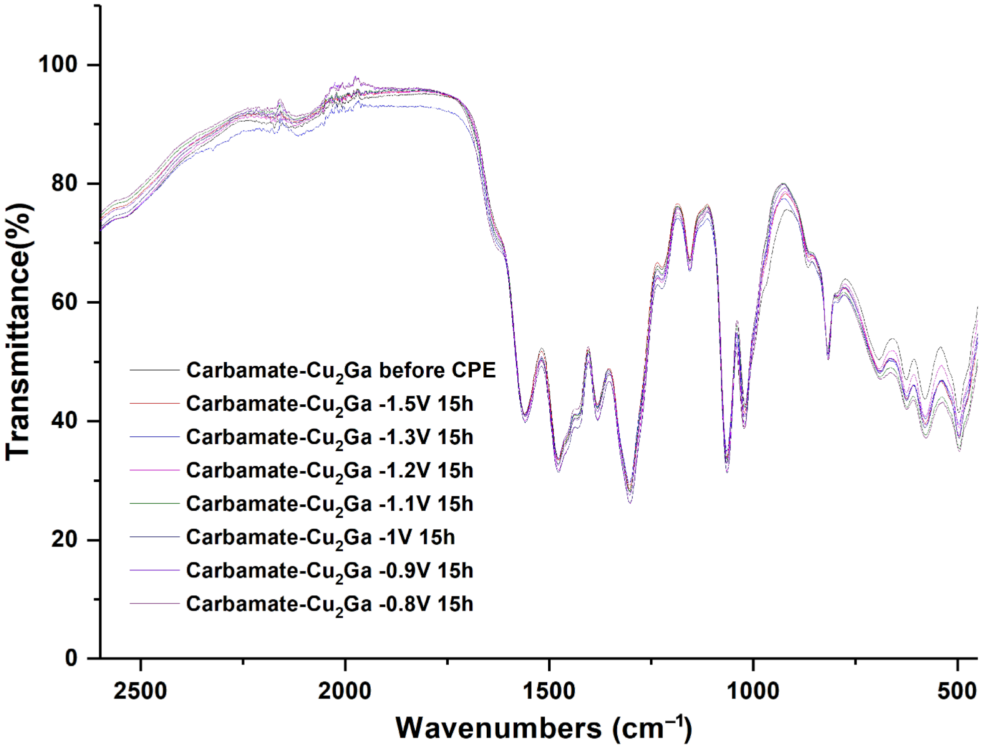

Figure S16. IR spectra of $\{SiCu_2GaW_9\}$ after controlled voltage electrolysis for 15 h. Cathode: 1 cm^2 carbon paper GDL from ink containing 80 wt% $\{SiCu_2GaW_9\}$, 10 wt% Aemion ionomer and 10 wt% carbon black. Anode: IrO_2 on Pt-Ti felt separated by a glass frit as counter electrode. Catholyte:1 mL PEI (molecular weight 1 M), 3 mL H_2O, 2 mL Na_7DTPMP 25 % (w/v) in H_2O and 0.8 M Na_2SO_4•10H_2O treated for 20 min under a flow of CO_2. Anolyte was Na_7DTPMP 25 % (w/v) in H_2O and 0.8 M Na_2SO_4•10H_2O. Nafion 107 membrane, room temperature.

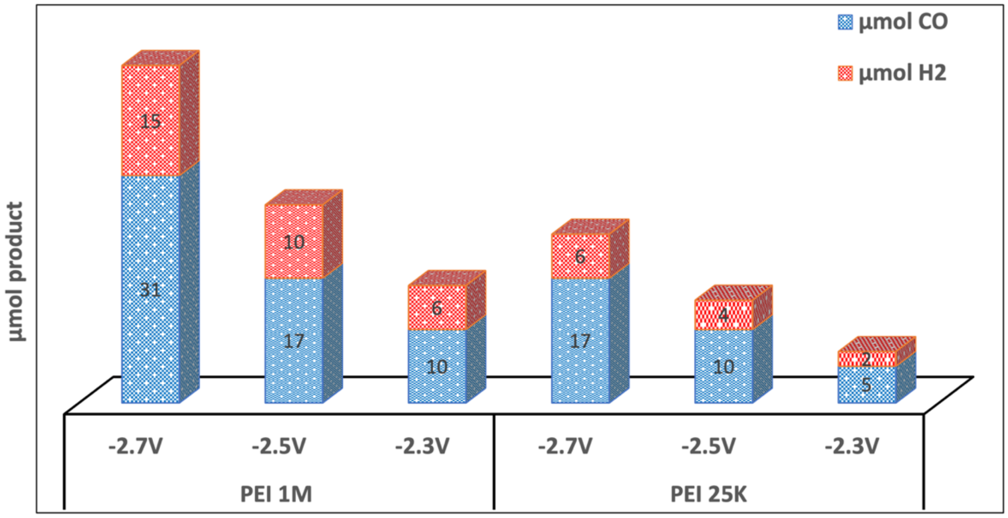

Figure S17. Controlled voltage electrolysis comparing PEI (1 M) and PEI (25 K) in an electrolyzer. Cathode: 1 cm^2 carbon paper GDL from ink containing 80 wt% $\{SiCu_2GaW_9\}$, 10 wt% Aemion ionomer and 10 wt% carbon black. Anode: IrO_2 on Pt-Ti felt separated by a glass frit as counter electrode. Catholyte:1 mL PEI (molecular weight 1 M), 3 mL H_2O, 2 mL Na_7DTPMP 25 % (w/v) in H_2O and 0.8 M Na_2SO_4•10H_2O treated for 20 min under a flow of CO_2. Anolyte was Na_7DTPMP 25 % (w/v) in H_2O and 0.8 M Na_2SO_4•10H_2O. Nafion 107 membrane, room temperature, time – 15 h.

Table S1. Faradaic efficiencies, current densities, product distributions and charge using three catalysts, two polyethylenimines and at three cell voltages.

Catalyst	Voltage	PE, MW	CO, μmol	H_2, μmol	F.E. (%)	J, mA/cm^2	Q, C
{$SiCu_2GaW_9$}	2.7	1000000	31	15	50.1	1.21	18.1
{$SiCu_2GaW_9$}	2.7	25000	11	7	23.1	1.04	15.6
{$SiCu_2GaW_9$}	2.5	1000000	17	10	40.0	0.90	13.5
{$SiCu_2GaW_9$}	2.5	25000	7	3	13.2	1.01	15.1
{$SiCu_2GaW_9$}	2.3	1000000	10	6	16.4	1.30	19.5
{$SiCu_2GaW_9$}	2.3	25000	3	1	5.1	0.91	13.6
{$SiCuFeGaW_9$}	2.7	1000000	36	12	44.2	1.45	21.7
{$SiCuFeGaW_9$}	2.7	25000	16	6	26.7	1.09	16.4
{$SiCuFeGaW_9$}	2.5	1000000	18	8	27.7	1.25	18.8
{$SiCuFeGaW_9$}	2.5	25000	11	4	19.9	1.01	15.1
{$SiCuFeGaW_9$}	2.3	1000000	0	0	0	0.06	0.9
{$SiCuFeGaW_9$}	2.3	25000	0	0	0	0.05	0.7
{$P_2Ni_4W_{18}$}	2.7	1000000	18	20	41.8	1.21	18.2
{$P_2Ni_4W_{18}$}	2.7	25000	12	15	32.5	1.11	16.6
{$P_2Ni_4W_{18}$}	2.5	1000000	4	6	12.0	1.11	16.7
{$P_2Ni_4W_{18}$}	2.5	25000	7	11	23.1	1.04	15.6
{$P_2Ni_4W_{18}$}	2.3	1000000	0	0	0	0.04	0.6
{$P_2Ni_4W_{18}$}	2.3	25000	0	0	0	0.06	0.9
